# The effectiveness and safety of botulinum toxin injections for the treatment of sialorrhea with Parkinson's disease: a systematic review and meta-analysis

**DOI:** 10.1186/s40360-023-00694-7

**Published:** 2023-10-12

**Authors:** Chun-Lan Yang, Jia-Peng Huang, Ying-chao Tan, Ting-ting Wang, Han Zhang, Yun Qu

**Affiliations:** 1 Department of Rehabilitation Medicine, West China Hospital, Sichuan University, Chengdu, Sichuan 610041 PR China; 2 Minda Hospital of Hubei Minzu University, Enshi, Hubei 445000 PR China; 3 Key Laboratory of Rehabilitation Medicine in Sichuan Province, West China Hospital, Sichuan University, Chengdu, Sichuan 610041 PR China; 4 Enshi Prefecture Central Hospital, Enshi, Hubei 445000 PR China

**Keywords:** Sialorrhea, Parkinson disease, Botulinum toxins, Safety

## Abstract

**Background:**

Botulinum toxin (BoNT) injection is an important adjunctive method to treat sialorrhea. The purpose of this systematic review was to analyze the effect and safety of BoNT injections in the intervention of sialorrhea with Parkinson’s disease (PD).

**Methods:**

We searched PubMed, Web Of Science (WOS), Scopus, Cochrane CENTRAL, and Embase from inception until April 2022. Randomized controlled trials or randomized crossover trials comparing BoNT with placebo in sialorrhea with PD were eligible. PRISMA guidelines were used to carry out the meta-analysis. The Drooling Severity Frequency Scale (DSFS) score and the number of adverse events (AEs) were the primary and secondary outcomes, respectively. Standardized mean differences (SMDs) and risk differences (RDs) are used to express continuous and categorical outcomes, respectively. Heterogeneity among these studies was evaluated using I^2^ tests. We used the GRADE tool to assess the certainty of evidence (COE).

**Results:**

Eight articles involving 259 patients compared BoNT injections with a placebo for PD with sialorrhea. This meta-analysis showed a significant reduction in DSFS scores between BoNT injections and placebo (SMD=-0.98; 95% CI, -1.27 to 0.70, *p*<0.001; COE: high). This meta-analysis showed a significant difference in AEs between BoNT injections and placebo (RD=0.15; 95% CI, 0.05 to 0.24, *p*=0.002; COE: low).

**Conclusions:**

The pooled results suggest that BoNT injections have some effect on DSFS scores with sialorrhea caused by PD. There are also mild adverse events, which generally recover within a week or so. The results indicate that BoNT injection is one of the treatments for sialorrhea caused by PD, but we need to pay attention to adverse events. In addition, the follow-up time was extended to observe oral hygiene, ulceration or dental caries, and digestive function.

**Trial registration:**

Our review protocol was registered on PROSPERO (42021288334).

**Supplementary Information:**

The online version contains supplementary material available at 10.1186/s40360-023-00694-7.

## Background

Salivation, also known as drooling, occurs when there is excess saliva in the mouth over the edge of the lips [1, 2]. This is normal in infants, but drooling gradually decreases as development matures and usually disappears at approximately 15 to 18 months [3]. Sialorrhea can cause embarrassment, isolation, depression, and skin infection and can lead to pneumonia because saliva is pooled in the mouth. Many approaches, such as physiotherapy, aspiration, medication, radiotherapy, and surgical intervention, have been used to manage sialorrhea [4]. However, these are symptomatic treatments, and there is no universally accepted treatment.

The advent of Botulinum toxin (BoNT) injections seems very promising. BoNT was initially thought to be the cause of food poisoning [5]. At the end of the 18th century, some disease outbreaks were linked to sausages in southwestern Germany [6]. In 1977, Allan Scott [7] first injected a patient with strabismus. Since then, BoNT has been increasingly used in clinical practice. Later, BoNT was also considered the treatment of choice for sialorrhea. Studies have shown that Rimabotulinum toxin B treatment of sialorrhea improves the unstimulated salivary flow rate and the clinical global impression of change [8]. A meta-analysis showed that BoNT types A and B significantly reduce salivation in patients with neurological disorders [9]. Growing research shows the potential of BoNT treatment for sialorrhea.

The effects of BoNT injections with sialorrhea have also been known for years, but their use was limited because of a lack of approval studies [10]. There are differences in the types and doses of BoNT used in different countries, such as the USA and Europe. OnabotulinumtoxinA and AbobotulinumtoxinA are off-label treatments for sialorrhea. A meta-analysis showed that the recommended level of BoNT treatment for sialorrhea in adults and children is different [11]. BoNT remains a controversial and attractive drug. This may be related to do with the etiology and mechanism of sialorrhea. Sialorrhea is more common in Parkinson's disease (PD), cerebral palsy, stroke, a side effect of medications, amyotrophic lateral sclerosis, schizophrenic and intellectual disease. Sialorrhea may be due to increased salivary secretion, failure to clear saliva in the mouth, and miscoordination of the oral-facial and palatoglossus muscles [2]. Therefore, the sialorrhea mechanism caused by each disease may be different, so the efficacy and adverse effects of BoNT treatment may be different.

As the population ages, the burden of PD is increasing, seriously affecting people's activities of daily living [12]. PD is the second most common neurodegenerative disorder, affecting approximately 1% of adults older than 60 years [13–16]. An estimated 6.2 million people worldwide suffer from PD [12, 17]. As research continues, our understanding of PD continues to evolve. Initially, we recognized that it was typically characterized by motor symptoms, such as rigidity, bradykinesia, and resting tremor [18]. Motor symptoms are key to diagnosing PD [19], but non-motor symptoms (NMS) of PD are common and often overlooked, such as sialorrhea, dementia, depression, and sleep disorders [20]. NMSs are prevalent in over 90% of PD patients [21], and there are more women than men [22]. NMSs are common in early PD, reflecting that the disease damages multiple systems [23]. Some NMSs have a greater impact on the quality of life of people with PD [24]. The most common in the early stages of PD is excessive saliva [23]. A large number of patients with PD undergo sialorrhea, ranging from 32% to 74% [25]. Studies show that more than 80% of patients with PD experience sialorrhea [26, 27]. We initially thought that Parkinson's patients had excessive salivary production, but studies have found that Parkinson's patients have less salivary production than normal people [28–30]. Sialorrhea can be caused by excessive salivation, difficulty swallowing, or both [29, 31]. Some studies have shown that sialorrhea is thought to be due to dysphagia, which reduces the effective removal of saliva rather than excessive drooling [32]. Some researchers have classified sialorrhea as gastrointestinal dysfunction or autonomic dysfunction [33, 34]. The pathogenesis of sialorrhea in PD remains controversial.

Currently, injections of BoNT reduce salivary production by blocking acetylcholine in the corresponding glands. A systematic review showed that BoNT is an effective method for treating sialorrhea with PD [35, 36]. However, subjects with sialorrhea caused by neurological diseases other than Parkinson's disease were included in this study, and a meta-summary analysis was not performed. There was also a study that reported no statistical significance of BoNT in the treatment of sialorrhea caused by PD [37]. There was also no analysis of the safety of BoNT in treating the saliva of PD patients. To compare the results of prior studies of BoNT injection, we included only randomized controlled trials and crossover trials to meta-analyze the efficacy and safety of BoNT in the treatment of sialorrhea in PD.

## Methods

### Protocol and registration

This meta-analysis was conducted according to the 2020 PRISMA statement [38]. Our review protocol was registered on PROSPERO (42021288334).

### Search strategy

We conducted a systematic search of the literature to identify all studies reported in English from five different databases: PubMed, Scopus, Cochrane CENTRAL, Embase, and Web of Science, up to April 2022. We also scanned at* Google Academic and clinicaltrials.gov*. We reviewed a randomized, placebo-controlled, and crossover trial of BoNT injection for patients who were diagnosed with sialorrhea and PD. The keywords we used were Botulinum Toxins, abobotulinum toxin A OR incobotulinum toxin A OR rimabotulinum toxin B, and sialorrhea. The search strategies for each database are described in Supplement S1.

### Eligibility criteria and study selection

We used Endnote software for literature management. First, two researchers (YCL and TYC) scanned the titles and abstracts to screen out potential studies and then read the full text to determine the final studies that met the criteria. Any disagreements were resolved by a third author (HJP).

Our inclusion criteria were as follows: 1) randomized controlled trial (RCT) or randomized crossover trial; 2) comparison of botulinum toxin with placebo; and 3) sialorrhea caused by PD. Excluded studies were as follows: 1) studies on patients enrolled in other neurological conditions except for PD; 2) non-English language published.

### Data extraction and outcome measures

Data were independently extracted from eligible studies by two authors (YCL and WTT). Extracted data were compared, and any discrepancies were resolved through discussion with the third author (HJP). Relevant data, such as study time, sample size, dosage, type, and outcome, were extracted from all included papers [39]. The primary outcomes were the Drooling Severity and Frequency Scales (DSFS) scores [40]. The secondary outcomes were adverse events (AEs), which were reported during the study. Data were collected using standard spreadsheets (Excel). If any information was unclear, we contacted the author to provide more detailed data.

### Statistical analysis

We used inverse-variance and fixed-effects models to perform the meta-analysis in Review Manager (version 5.4; Cochrane Collaboration, Oxford, UK). The results for all outcomes were calculated using the change between the control group and the placebo group. We used SMDs and RDs to represent continuous and categorical results, respectively, as well as the 95% CIs. The I^2^ was reported as a measure of heterogeneity. The level of significance was set at *p* < 0.05. To provide clinical evidence, we divided these clinical trials into subgroups of BotoxA and BotoxB to investigate the efficacy and safety of BotoxA and BotoxB, respectively.

### Assessment of risk of bias

The methodological quality of each study was independently assessed by the two reviewers (YCL and TYC) using the Cochrane Collaboration risk of bias method [37]. Disagreements were resolved through consultation with the third reviewer (HJP), if necessary. This instrument evaluates seven domains: random sequence generation, allocation concealment, performance bias, detection bias, attrition bias, reporting, and other biases. The overall risk of bias is low if the risk in all study areas is low. If there is a high risk in one region or uncertainty in multiple regions, the overall risk of bias is higher. The remaining studies were considered to have some concerns about the overall risk of bias.

### Reporting bias assessment

We planned to generate funnel plots for the meta-analysis. If the forest map is asymmetric, we plan to review the characteristics of the trial to assess whether the asymmetry may be due to publication bias or other factors, such as methodological or clinical trial heterogeneity. To assess the reporting bias, we compared the results specified in the trial protocol with those reported in the corresponding trial publication. If tracking protocols were not available, we compared the results reported in the methods and results section of tracking publications.

### Certainty assessment

We used the GRADE approach to assess the quality of evidence by importing data from RevMan 5.4 into a GRADE profile. The rating aspects were study limitations, inconsistency, indirectness, imprecision, and publication bias [41].

### Patient and public involvement

Patients and/or the public were not involved in the design, conduct, reporting, or dissemination plans of this research**.**

## Results

### Selection and characteristics of studies

**Fig. 1:**
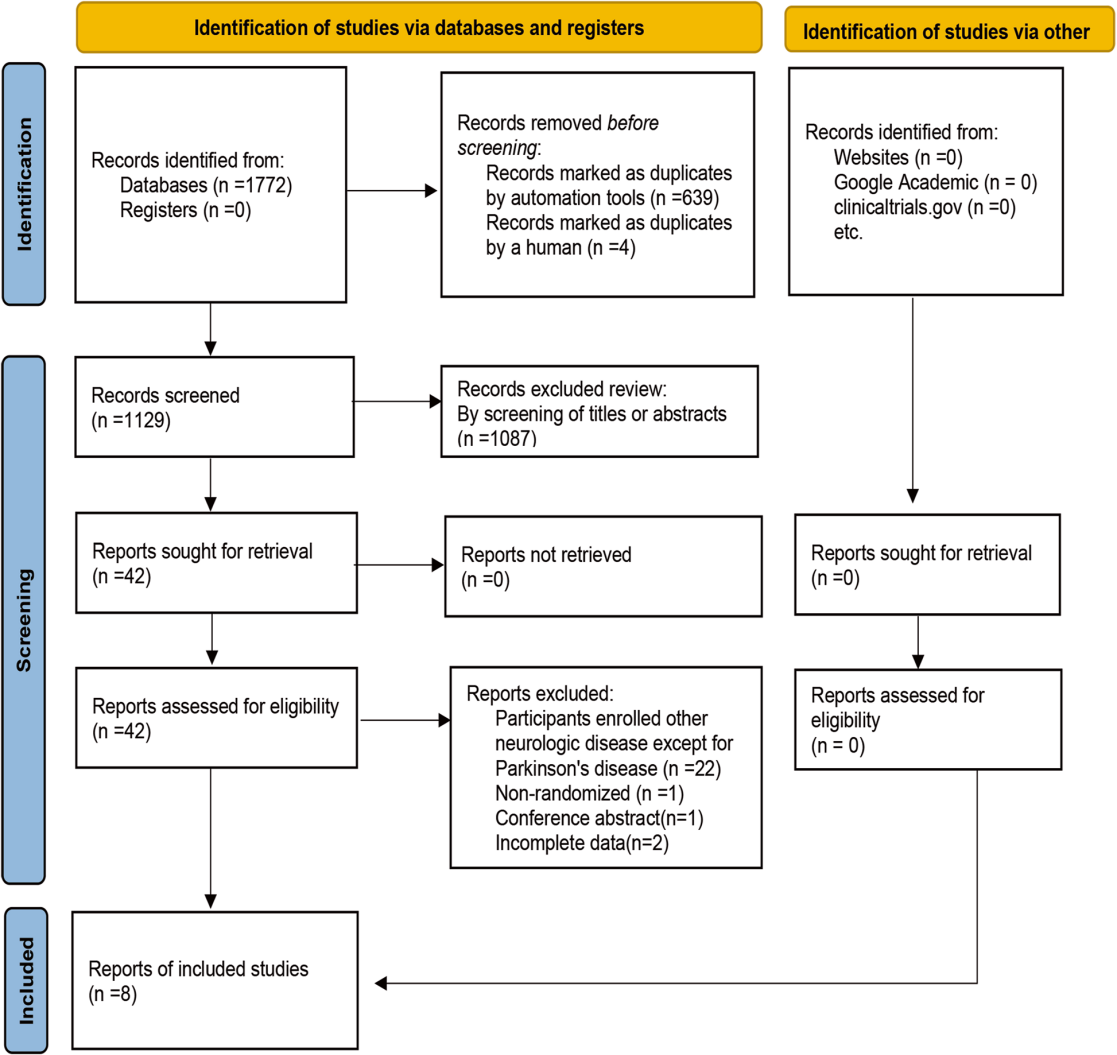
Flow chart of the literature search process

Figure 1 is a flow chart of the selection process for the study. A total of 1772 studies were retrieved from the database, and 643 duplicate references were excluded by Endnote. After reviewing the titles or abstracts, another 1,087 studies were excluded because they were reviews or irrelevant. The full text of the remaining 42 studies was retrieved. The first screening excluded 23 studies that included neurological disorders and 11 non-randomized controlled trial studies. Eight studies were included [37, 40, 42–47]. One study did not include FSES scores in the results but evaluated saliva composition and adverse events [47]. Two studies could not obtain post-injection data for the experimental and control groups, and the authors could not be contacted [40, 46]. One study was a conference abstract [37]. One study used indirect data derived from reviews by others [46]. One study used three control doses, all of which were statistically significant compared with placebo [45]. Adverse events were reported in all studies. The characteristics of the included studies are detailed in Table 1.

**Table 1:** Characteristics of included studies

Study Year Country	No. of patients	Mean age (years) (mean ± SD)	Gender (M/F)		Type	Outcome	Location Method	Adverse events(n)	Doses (U)
Mancini [40] 2003 Italy	BoNT: 10 Placebo: 10	69.6 ± 6.1 69.1 ± 6	6/4 5/5		A	DSFS	Ultrasound guidance	No reported (0)	225
Ondo [42] 2004 USA	BoNT: 8 Placebo: 8	68.8± 10.2 72.0± 13.0	13/3		B	DSFS Drooling Rating Scale Scintigraphy (10^3^ CT/MCI) Dysphagia Scale	Anatomic markers	Dry mouth (3) Worsened gait (2) Diarrhea (1) Neck pain (1)	2500
Lagalla [43] 2006 Italy	BoNT: 16 Placebo:16	69.4 ± 5.5 70.5 ± 5.5	13/3 11/5	A		VAS-D score UPDRS-ADL drooling Item	Anatomic markers	Swallowing (1)	100
Lagalla [44] 2009 Italy	BoNT: 18 Placebo: 18	73.1 ± 5.8 70.8 ± 6	14/4 12/6	B		DSFS UPDRS-ADL Drooling Item	Anatomic markers	Swallowing (3)	4000
Chinnapongse [45] 2012 USA	BoNT: 14 Placebo: 15	67.6±7.07 71.2±11.64	12/2 13/2	B		DSFS Drooling Rating Scale Visual analog scale	Anatomic markers	Dry mouth (2) serious (0)	1500
Chinnapongse2 [45] 2012 USA	BoNT: 12 Placebo: 15	71.8± 8.17 71.2±11.64	12/12 13/2	B		DSFS Drooling Rating Scale Visual analog scale	Anatomic markers	Dry mouth (3) Serious (1)	2500
Chinnapongse3 [45] 2012 USA	BoNT: 13 Placebo:15	74.1±5.47 71.2±11.64	11/2 13/2	B		DSFS Drooling Rating Scale Visual analog scale	Anatomic markers	Dry mouth (1) Serious (1)	3500
Narayanaswami [37] 2015 USA	BoNT: 9 Placebo:9	86±0.92	6/3	A		DSFS Saliva weight	No detailed	Difficulty chewing (1) Thick saliva (1)	100
Narayanaswami [46] 2016 USA	BoNT: 9 Placebo: 9	64.7 ± 4.8 70.8± 12.3	6/3	A		DSFS	Anatomic markers	Chewing (1)	100
Janne [47] 2018 Estonia	BoNT: 12 Placebo:13	57.7 ± 9.6 63.7 ± 8.1	9/3 7/6	A		Resting saliva formation time Amount of 5-min collected saliva PH	Ultrasound guidance	Saliva thickening (1)	250

*BoNT* Botulinum toxin, *DSFS* Drooling Severity and Frequency scales, *VAS* Visual Analog Scale, *D* Drooling frequency

### Study quality

**Fig. 2:**
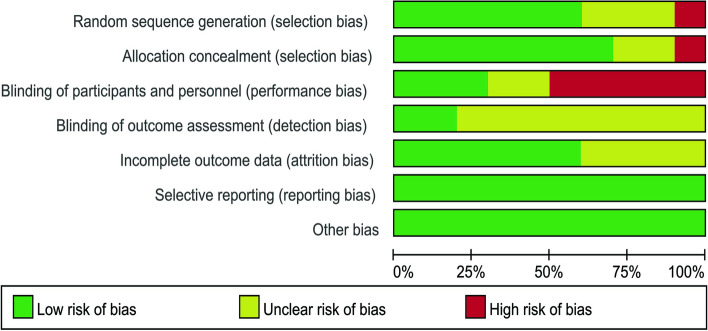
Risk of bias graph

**Fig. 3:**
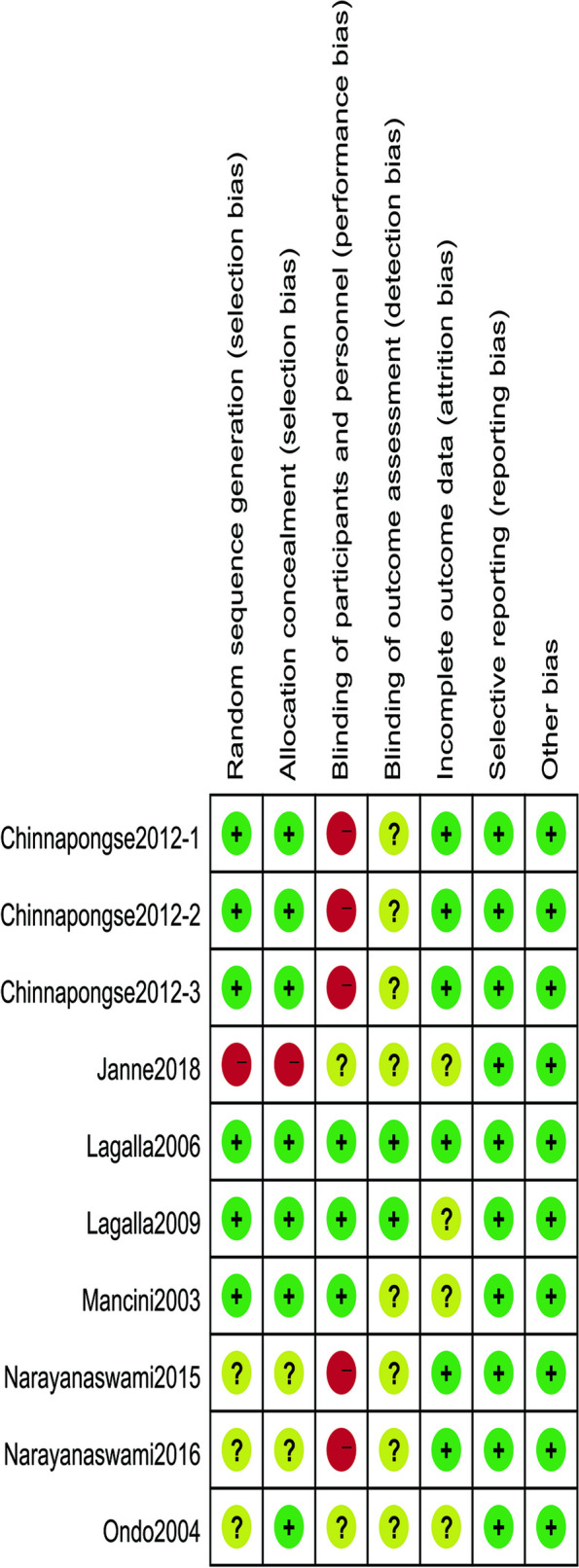
Risk of bias summary

We evaluated the literature quality of the study using the risk assessment tool of RevMan 5.4 software, as shown in Figs. 2 and 3. In selection bias, six studies were assessed as low, three were rated as unclear due to lack of detail [37, 42, 46], and one study did not explicitly report the use of randomization [47]. Allocative concealment was classified as unclear in two studies [37, 46], and one study was unlikely to use allocative concealment [47]. Because it was not possible to conduct blind testing of practitioners and participants considering dose differences and crossover trials, three studies were judged to be at high risk of performance bias in performance and detection bias [37, 45, 46]. Only two studies were blinded [43, 44], and other studies were unclear in terms of blinding outcome assessment. Four studies [40, 42, 44, 47] did not provide enough information to judge the risk of bias with incomplete outcome data. Selective reporting is not possible for all studies. There were no other risks of bias in any of the studies.

### Outcome of DSFS

**Fig. 4:**
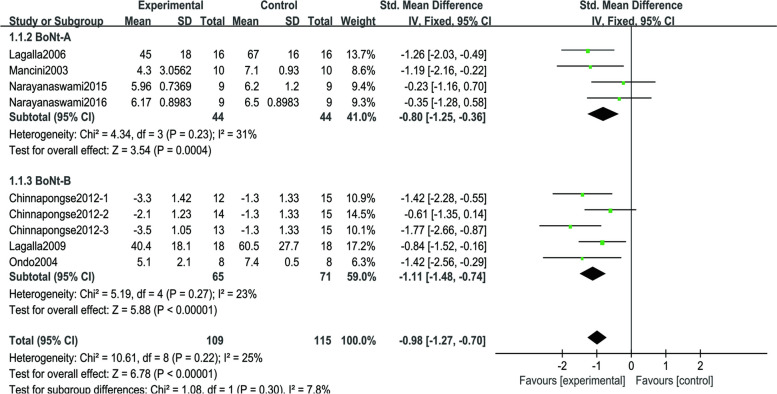
Forest plot of comparison: DSFS. DSFS: Drooling severity frequency scale

**Fig. 5:**
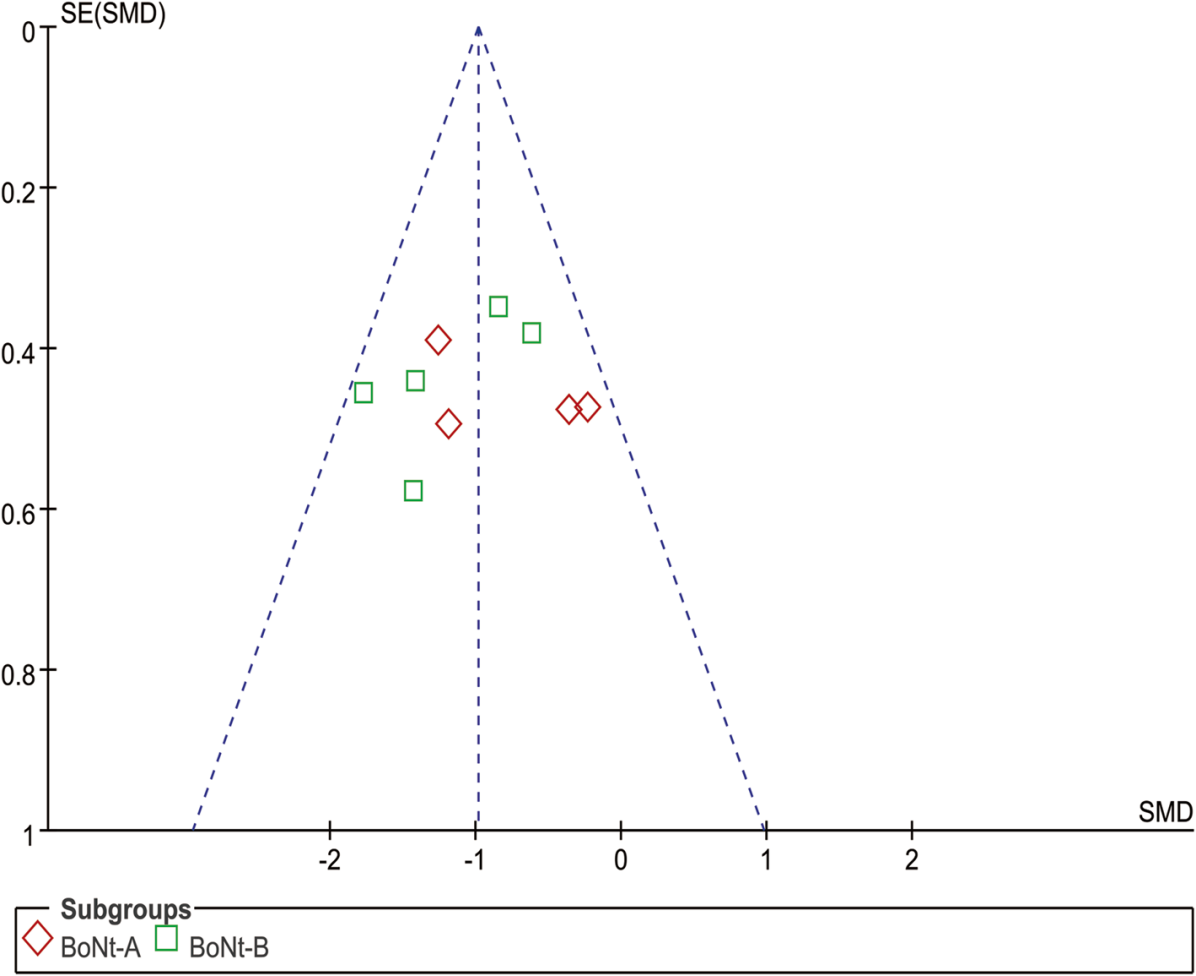
Funnel plot of comparison: DSFS. DSFS: Drooling severity frequency scale

Nine studies involving 224 patients reported information about DSFS outcomes. Two studies cited data from others because they did not report outcomes after treatment in the BoNT injection group and the placebo group [37, 40, 46]. In one study, we used the median instead of the mean [46]. As depicted in Fig. 4, this meta-analysis showed a significant reduction in DSFS scores between BoNT injections and placebo (SMD=-0.98; 95% CI, -1.27 to 0.70, *p*<0.001; COE: high); no significant heterogeneity was detected across the nine studies (*p* = 0.22, I^2^=25%). In terms of subgroup analysis, there was no statistically significant difference between the two groups, indicating that there was no significant difference in effect between type A and Type B. Publication bias is unlikely from the funnel plot (Fig. 5).

### Outcome of adverse events

**Fig. 6:**
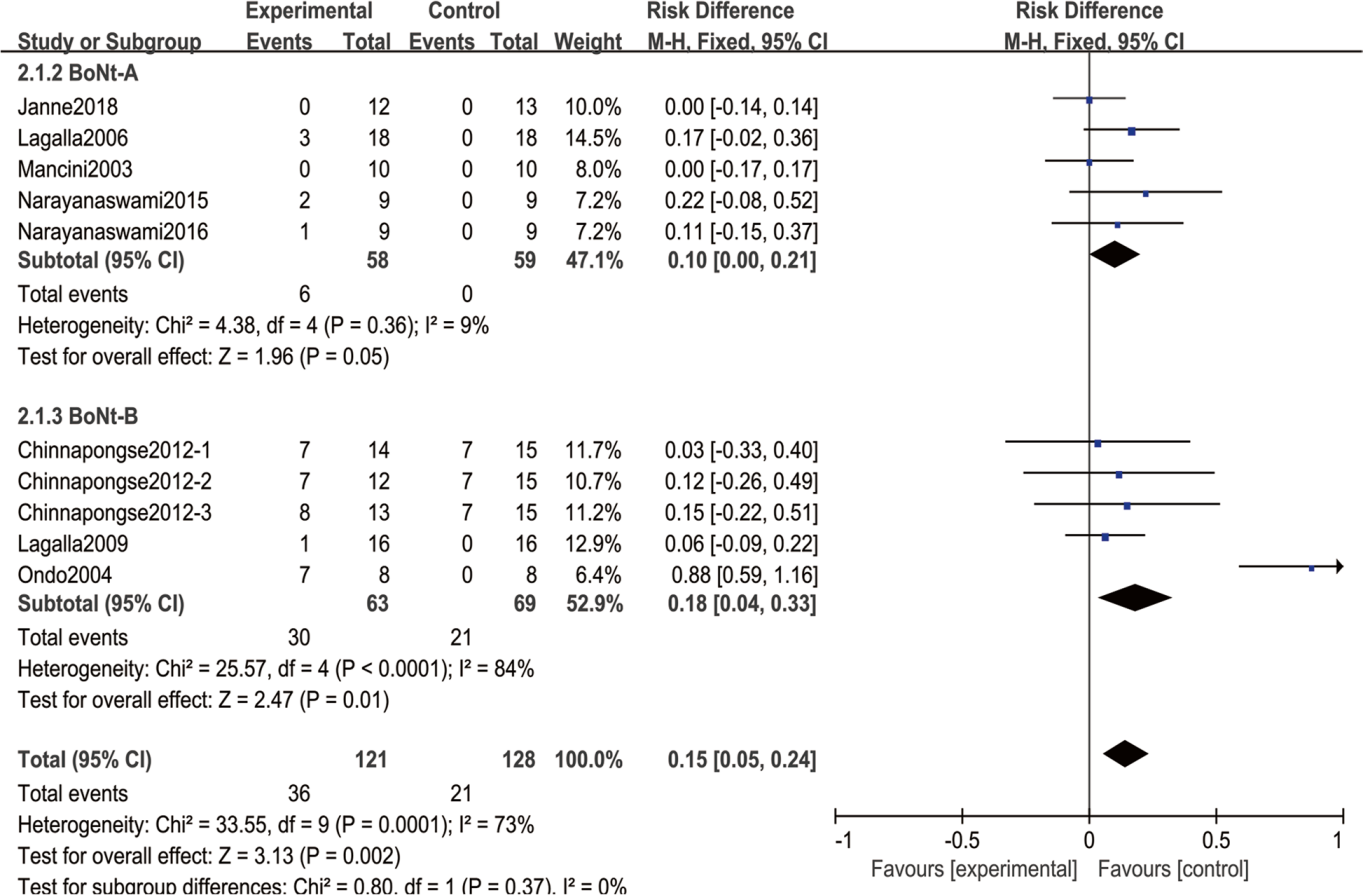
Forest plot of comparison: adverse events

**Fig. 7:**
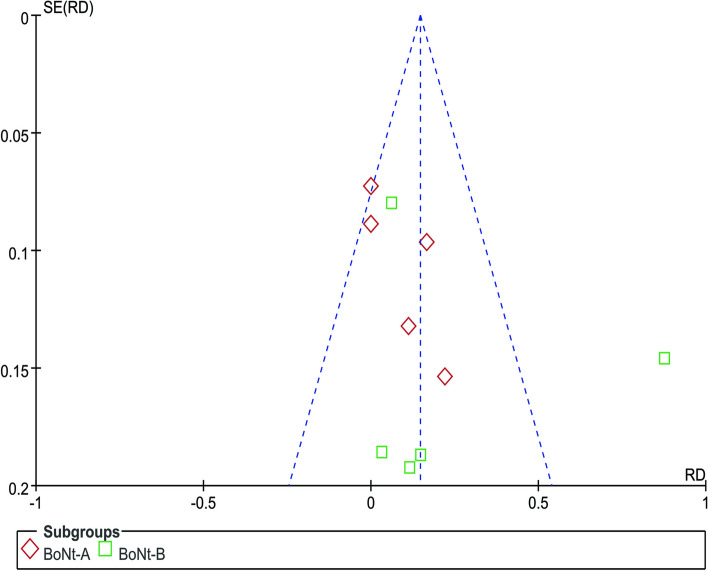
Funnel plot of comparison: adverse events

AEs were counted in all ten trials involving subjects. Figure 6 shows a difference in AEs between BoNT injections and placebo (RD=0.15; 95% CI, 0.05 to 0.24, *p*=0.002; COE: low); The severity of most treatment-related AEs was mild to moderate and self-limited. Patients taking BoNT had dry mouth significantly more often than those taking placebo, and other AEs included neck pain, diarrhea, and worsened gait. Two botulinum toxin-B subjects and three placebo subjects experienced at least one serious AE [45], atrial fibrillation, and urosepsis. None were deemed related to the study drug, and all events were resolved. In terms of subgroup analysis, there was no statistically significant difference between the two groups (*p* = 0.37, I^2^=0%). During the heterogeneity analysis, the heterogeneity of one study was obvious, and after excluding this study [42], the heterogeneity was not significant across the nine studies (*p* > 0.05, I^2^=0%). This study reported significantly more adverse events than other studies, but we found no systemic design flaws in this study, and there is no reason to exclude it. Two studies reported zero adverse events, which is also far behind the others [40, 47]. Publication bias is likely from the funnel plot (Fig. 7).

## Discussion

The results provide a new understanding of the effectiveness of BoNT injection on sialorrhea with PD.

Compared with a placebo, BoNT significantly reduced the DSFS of sialorrhea in PD patients. The recommended evidence level is high in terms of FESF score improvement. Consider that physical therapy for PD is short-term benefits and time consuming [48]. Other oral anticholinergics may have significant side effects. Therefore, BoNT may be a better choice. If the patient is well informed, BoNT can be recommended for salivary patients with PD. However, the small sample size and the occurrence of coupling events should arouse our attention. The included studies measured results after one month, and one study measured results within one week [40]. The different dosages of all of these studies also contributed to greater heterogeneity in the comparison. One study did not show statistically significant changes in DSFS scores before and after treatment, but its sample size was small [37]. There were also three studies that did not directly publish the DSFS scores of the BoNT and placebo groups after treatment, which could also lead to errors in the pooled analysis [37, 40, 46].

Injection positioning includes ultrasound guidance, and anatomic positioning [49]. *Wolfgang H. Jost* [50] believes that ultrasonic guidance is safer and that the operation speed is not slowed down. Amanda *Amrita Lakraj* [2] and colleagues used nine points of refraction on one side of the face. However, there is a lack of comparison of therapeutic effects with different localization methods. The 200 U Botulinum Toxin A group had the largest decrease in saliva before 24 weeks compared to 100 U [51]. There are few studies on the relationship between dose and efficacy of BoNT.

We are very concerned about the adverse effects of BoNT injection for the treatment of sialorrhea in PD. The incidence of adverse events in the treatment group was higher than that in the placebo group, but the adverse events were mild. And the heterogeneity between studies was high. Dry mouth was the most common adverse event, with worsening gait, diarrhea, difficulty swallowing, neck pain, and weak chewing reported, with recovery time ranging from one to six weeks. Some studies have serious adverse events in both the treatment and placebo groups, although the final analysis may not be relevant to the treatment. Atrial fibrillation, urinary sepsis, and rectal bleeding were more serious adverse events in the treatment group. Congestive heart failure, dyspnea, pneumonia, and other symptoms occurred in the placebo group [45]. BoNT injection is a minimally invasive procedure, and it remains to be seen whether the procedure itself will affect the patient. Although the conclusion is not related to treatment, it should arouse our attention.

Although both types A and B are used in salivary therapy, they both work by blocking acetylcholine [27]. Saliva secretion is reduced, and the patient's sialorrhea symptoms improve. Saliva is secreted at 1 to 1.5 L per day and plays an important role in the oral, digestive, immune, and internal environmental systems. If you reduce the secretion of saliva, these areas will have an impact. Treatment with botulinum toxin can worsen dry mouth, dysphagia, gait, and weakness [52]. Studies have shown that long-term use of BoNT injection can reduce the size of salivary glands as measured by ultrasound [53]. We should also care about oral health and increase the frequency of dental visits [49]. Many animal studies show that repeated injections of toxins can lead to cumulative muscle atrophy [54]. Follow-up should be extended to determine whether prolonged treatment will lead to increased dysphagia and oral health deterioration. *Omar R. Tumilasci’s* [52] research indicated that basal and reflex salivary secretion is reduced in PD. Sialorrhea in PD is caused by dysphagia rather than excessive salivation. Whether we can treat salivation by improving swallowing function with BoNT instead of injecting it into salivary glands. In the future, we should continue to study the mechanism of botulinum toxin in the treatment of sialorrhea. There are still many problems to be discussed in the treatment of Parkinson's sialorrhea.

### Strengths and limitations of this study

Sialorrhea can be caused by various diseases. This is the first systematic review and meta-analysis to analyze sialorrhea in PD patients. The data from some small clinical randomized controlled trials and crossover trials are pooled into larger samples to provide evidence for clinical application. At the same time, the adverse events were objectively evaluated to provide a basis for the treatment of PD sialorrhea with botulinum toxin. However, there are some shortcomings: 1) Fewer studies were included, and the number of patients included in some studies was small; 2) The evaluation results were mainly subjective, with less objective evaluation; 3) Long-term adverse reactions were not followed up; 4) This study does not negate the use of botulinum toxin in Parkinson's sialorrhea, but it will allow more researchers to study the mechanism and pay attention to the side effects. 5) The effect of BoNT on sialorrhea may be related to the disease itself, and our study is only a small part of many diseases.

## Conclusion

There was no statistically significant difference in efficacy between Botulinum toxin A and Botulinum toxin B. Many studies now show that Botulinum toxin has value in treating Parkinson-related saliva, while others suggest that it is less effective. The COE of efficacy was high. However, the level of evidence for safety is low. No serious adverse events directly related to botulinum toxin have been reported. But we also have to pay attention to adverse events. However, there were significant differences in mild adverse events. Therefore, larger samples and more scientifically designed randomized controlled trials are needed to explore the safety of botulinum toxin as a potential alternative treatment for sialorrhea caused by PD. We should also pay attention to the dose, duration, and duration of action of botulinum toxin. More attention should be given to the pathological mechanism of sialorrhea in PD.

### Supplementary Information

**Additional file 1.** 

## Data Availability

All data generated or analyzed during this study are included in this publication

## Abbreviations

AEs: adverse events
BoNT: botulinum toxin
COE: certainty of evidence
CI: Confidence interval
DSFS: Drooling severity frequency scale
GRADE: Grading of Recommendations, Assessment, Development, and Evaluation
NMS: Non-motor symptoms
PD: Parkinson's disease
PRISMA: the Preferred Reporting Items for Systematic reviews and Meta-Analyses
RCTs: randomized controlled trials
RDs: Risk differences
SMD: Standardized mean difference
WOS: Web Of science
