# Supplementary material for: The effectiveness and safety of botulinum toxin injections for the treatment of sialorrhea with Parkinson's disease: a systematic review and meta-analysis
Source: BMC Pharmacol Toxicol. 2023 Oct 12;24:52. doi: 10.1186/s40360-023-00694-7 (PMC10571401; doi:10.1186/s40360-023-00694-7)
Supplement: Supplementary file 1 — Additional file 1. [file 40360_2023_694_MOESM1_ESM.pdf]

	Search Strategy
PubMed	(("botulinum toxins"[MeSH Terms] OR "neurotoxins botulinum"[Title/Abstract] OR "botulinum toxin"[Title/Abstract] OR "toxin botulinum"[Title/Abstract] OR "clostridium botulinum toxins"[Title/Abstract] OR "toxins clostridium botulinum"[Title/Abstract] OR "botulinum neurotoxin"[Title/Abstract] OR "neurotoxin botulinum"[Title/Abstract] OR "Botulin"[Title/Abstract] OR ("botulinum toxins, type a"[MeSH Terms] OR "clostridium botulinum a toxin"[Title/Abstract] OR "botulinum toxin a"[Title/Abstract] OR "toxin a botulinum"[Title/Abstract] OR "botulinum neurotoxin a"[Title/Abstract] OR "neurotoxin a botulinum"[Title/Abstract] OR "botulinum a toxin"[Title/Abstract] OR "toxin botulinum a"[Title/Abstract] OR "botulinum toxin type a"[Title/Abstract] OR "botulinum neurotoxin type a"[Title/Abstract] OR "clostridium botulinum toxin type a"[Title/Abstract] OR "Meditoxin"[Title/Abstract] OR "Botox"[Title/Abstract] OR "Neuronox"[Title/Abstract] OR "Oculinum"[Title/Abstract] OR "onabotulinumtoxin a"[Title/Abstract] OR "Vistabel"[Title/Abstract]) OR ("abobotulinum toxin a"[Title/Abstract] OR "abobotulinumtoxin a"[Title/Abstract] OR "Dysport"[Title/Abstract] OR "azzalure"[Title/Abstract]) OR ("BoNT-A"[Title/Abstract] OR "NT-201"[Title/Abstract] OR "NT-201"[Title/Abstract] OR "NT201"[Title/Abstract] OR "bocouture"[Title/Abstract] OR "Xeomin"[Title/Abstract]) OR (("rimabotulinum"[All Fields] AND "toxin b"[Title/Abstract]) OR "NeuroBloc"[Title/Abstract] OR "botulinum b toxin"[Title/Abstract] OR "botulinum toxin type b"[Title/Abstract] OR "botulinum neurotoxin b"[Title/Abstract] OR "clostridium botulinum b toxin"[Title/Abstract] OR "Myobloc"[Title/Abstract])) AND ("sialorrhea"[MeSH Terms] OR "Hypersalivation"[Title/Abstract] OR "Drooling"[Title/Abstract])) AND ((clinicaltrial[Filter] OR randomizedcontrolledtrial[Filter]) AND (english[Filter]))
WOS	#1Sialorrhea (Topic) or Hypersalivation (Topic) and Drooling (Topic)<br>#2Botulinum Toxin* (Abstract) or Neurotoxin*, Botulinum (Topic) or Toxin, Botulinum (Topic) or Clostridium botulinum Toxins (Topic) or Toxins, Clostridium botulinum (Topic) or Botulinum Neurotoxin (Topic) or Botulin (Topic) or onabotulinumtoxinA (Topic) or Clostridium botulinum A Toxin (Topic) or Botulinum Toxin A (Topic) or Toxin A, Botulinum (Topic) or Botulinum Neurotoxin A (Topic) or Neurotoxin A, Botulinum (Topic) or Botulinum A Toxin (Topic) or Toxin, Botulinum A (Topic) or Botulinum Toxin Type A (Topic) or Botulinum Neurotoxin Type A (Topic) or Clostridium Botulinum Toxin Type A (Topic) or Meditoxin (Topic) or Botox (Topic) or Neuronox (Topic) or Oculinum (Topic) or Vistabex (Topic) or Onabotulinumtoxin A (Topic) or Vistabel (Topic) or abobotulinumtoxinA (Topic) or abobotulinum toxin A (Topic) or abobotulinumtoxin A (Topic) or Dysport (Topic) or azzalure (Topic) or incobotulinumtoxinA (Topic) or BoNT-A (Topic) or NT 201 (Topic) or NT*201 (Topic) or bocouture (Topic) or Xeomin (Topic) or rimabotulinum toxin B (Topic) or rimabotulinumtoxin B (Topic) or NeuroBloc (Topic) or botulinum B toxin (Topic) or botulinum toxin type B (Topic) or botulinum neurotoxin B (Topic) or Clostridium botulinum B toxin (Topic) or Myobloc (Topic)<br>#3(#2) AND #1
Scopus	((TITLE-ABS-KEY(Sialorrhea) OR TITLE-ABS-KEY(Hypersalivation) OR TITLE-ABS-KEY(Drooling))) AND ((TITLE-ABS-KEY(Botulinum Toxin*) OR TITLE-ABS-KEY(Neurotoxin*, Botulinum) OR TITLE-ABS-KEY(Toxin, Botulinum) OR TITLE-ABS-KEY(Clostridium botulinum Toxins) OR TITLE-ABS-KEY(Toxins, Clostridium botulinum) OR TITLE-ABS-KEY(Botulinum Neurotoxin) OR TITLE-ABS-KEY(Botulin) OR TITLE-ABS-KEY(onabotulinumtoxinA) OR TITLE-ABS-KEY(Clostridium botulinum A Toxin) OR TITLE-ABS-KEY(Botulinum Toxin A) OR TITLE-ABS-KEY(Toxin A, Botulinum) OR TITLE-ABS-KEY(Botulinum Neurotoxin A) OR TITLE-ABS-KEY(Neurotoxin A, Botulinum) OR TITLE-ABS-KEY(Botulinum A Toxin) OR TITLE-ABS-KEY(Toxin, Botulinum A) OR

	TITLE-ABS-KEY(Botulinum Toxin Type A) OR TITLE-ABS-KEY(Botulinum Neurotoxin Type A) OR TITLE-ABS-KEY(Clostridium Botulinum Toxin Type A) OR TITLE-ABS-KEY(Meditoxin) OR TITLE-ABS-KEY(Botox) OR TITLE-ABS-KEY(Neuronox) OR TITLE-ABS-KEY(Oculinum) OR TITLE-ABS-KEY(Vistabex) OR TITLE-ABS-KEY(Onabotulinumtoxin A) OR TITLE-ABS-KEY(Vistabel) OR TITLE-ABS-KEY(abobotulinumtoxinA) OR TITLE-ABS-KEY(abobotulinum toxin A) OR TITLE-ABS-KEY(abobotulinu
Embase	#1sialorrhea:ti,ab,kw OR hypersalivation:ti,ab,kw OR drooling:ti,ab,kw<br>#2'botulinum toxin*':ti,ab,kw OR 'neurotoxin*, botulinum':ti,ab,kw OR 'toxin, botulinum':ti,ab,kw OR 'clostridium botulinum toxins':ti,ab,kw OR 'toxins, clostridium botulinum':ti,ab,kw OR 'botulinum neurotoxin':ti,ab,kw OR botulin:ti,ab,kw OR onabotulinumtoxina:ti,ab,kw OR 'clostridium botulinum a toxin':ti,ab,kw OR 'botulinum toxin a':ti,ab,kw OR 'toxin a, botulinum':ti,ab,kw OR 'botulinum neurotoxin a':ti,ab,kw OR 'neurotoxin a, botulinum':ti,ab,kw OR 'botulinum a toxin':ti,ab,kw OR 'toxin, botulinum a':ti,ab,kw OR 'botulinum toxin type a':ti,ab,kw OR 'botulinum neurotoxin type a':ti,ab,kw OR 'clostridium botulinum toxin type a':ti,ab,kw OR meditoxin:ti,ab,kw OR botox:ti,ab,kw OR neuronox:ti,ab,kw OR oculinum:ti,ab,kw OR vistabex:ti,ab,kw OR 'onabotulinumtoxin a':ti,ab,kw OR vistabel:ti,ab,kw OR abobotulinumtoxina:ti,ab,kw OR 'abobotulinum toxin a':ti,ab,kw OR 'abobotulinumtoxin a':ti,ab,kw OR dysport:ti,ab,kw OR azzalure:ti,ab,kw OR incobotulinumtoxina:ti,ab,kw OR 'bont a':ti,ab,kw OR 'nt 201':ti,ab,kw OR nt*201:ti,ab,kw OR bocouture:ti,ab,kw OR xeomin:ti,ab,kw OR 'rimabotulinum toxin b':ti,ab,kw OR 'rimabotulinumtoxin b':ti,ab,kw OR neurobloc:ti,ab,kw OR 'botulinum b toxin':ti,ab,kw OR 'botulinum toxin type b':kw OR 'botulinum neurotoxin b':ti,ab,kw OR 'clostridium botulinum b toxin':ti,ab,kw OR myobloc:ti,ab,kw<br>#3#1AND#2
CENTRAL	#1 MeSH descriptor: [Botulinum Toxins] explode all trees 2008<br>#2 MeSH descriptor: [Sialorrhea] explode all trees 114<br>#3 #1AND#2 in Trials, Clinical Answers
